# An observer tool to enhance learning of medical students during simulation training of cardiopulmonary resuscitation: a randomised controlled trial

**DOI:** 10.1186/s12909-024-05658-x

**Published:** 2024-07-03

**Authors:** Ammar Goulamhoussen, Caroline Havard, Benoit Gille, Bob François, Dan Benhamou, Antonia Blanié

**Affiliations:** 1 Centre de simulation LabForSIMS, Département de Recherche et Innovation Pédagogique en Santé, Faculté de médecine, Université Paris Saclay, Le Kremlin-Bicêtre, 94275 France; 2 Département d’Anesthésie-Réanimation Médecine Péri Opératoire, APHP, CHU Bicêtre, Le Kremlin Bicêtre, 94275 France; 3 CIAMS, Univ. Paris-Saclay, Université Paris-Saclay, Orsay Cedex, 91405 France; 4 CIAMS, Université d’Orléans, Orléans, 45067 France

**Keywords:** Simulation, Observer tool, Observational learning, Cardiopulmonary resuscitation, Education

## Abstract

**Background:**

Simulation training in cardiopulmonary resuscitation (CPR) is effective but active practice time is limited given the large number of students and the learning effect size remains small. To improve learning during observation, the use of an observer tool (OT) has been advocated. The aim was to assess the value of OT to improve medical students' learning outcomes during CPR simulation training.

**Methods:**

This prospective, randomized study took place during CPR training of medical students. The workshop targeted recognition of unconsciousness, absence of breathing, call for help, cardiac massage and defibrillation. Students practicing in dyads were randomized to use an OT (i.e., a checklist summarizing CPR skills and step-by-step actions) (OT +) or not (OT-) when observing others. At the end of the training, the global performance of the dyad was assessed by an evaluator using the OT checklist (primary outcome). The non-technical skills (NTS), chest compression quality, perceived improvement in knowledge and skills and knowledge score (MCQ) were also recorded.

**Results:**

The student dyads were included (OT + : *n* = 40 and OT-: *n* = 41). Immediately after training, the global performance was similar between the two groups: OT + : 24 [23—25] and OT-: 23 [21—24] (out of 25), *p* = 0.052. However, better learning of breathing assessment and cardiac massage performance, as well as a better knowledge score, were found in the OT + group. No significant difference was observed for NTS or perceived improvement in knowledge and skills. Satisfaction was higher in the OT- group.

**Conclusions:**

The use of an OT during CPR simulation did not show any pedagogical benefit on the global performance of medical students. However, a potential benefit was found for several important secondary outcomes. Further studies are needed to confirm these positive results.

**Supplementary Information:**

The online version contains supplementary material available at 10.1186/s12909-024-05658-x.

## Background

International scientific bodies advocate that everyone in the general population should learn to recognize cardiac arrest and manage first aid actions [1, 2]. Many teaching strategies have already been tested, but their effectiveness remains limited. Simulation training is an effective means to master cardiopulmonary resuscitation (CPR) skills [1–3]. Training in basic life support (BLS) is mandatory for French medical students. However, given the large number of students and the limited number and duration of training sessions, active practice time is limited, and they remain observers of their colleagues for a large part of the sessions.

The social learning theory proposed by Bandura and adapted to simulation states that vicarious learning occurs because from observing others one can get an idea of how behaviours are produced and then how to reproduce them [4]. Observation in simulation-based medical education may be effective [4–14] but a recent meta-analysis suggests that learning is more limited for an observer than for an active participant [5]. To increase the educational effects of simulation training when the learner is in the role of observer, some authors have proposed to use an observer tool (OT) allowing observers to analyse the progress of the task performed by their colleagues [4, 15–18]. OT are checklists in which key points to be achieved are listed in a consecutive manner. Studies using high-fidelity simulation have been performed in which OT were successfully used by residents during crisis management training in the operating room [16] but their educational usefulness is not well demonstrated.

The objective of this study was to assess whether the use of an OT can improve the learning process during CPR training for undergraduate medical students.

## Methods

### Study description

This prospective and randomised study was conducted at the simulation centre of a medical school (Paris Saclay University). This study was performed after ethical committee approval (SFAR, CERAR: IRB 00010254—2021 – 225). The trial had been registered on ClinicalTrials.gov (Identifier: NCT05187299; 11/01/2022). The study was carried out with the use of the CONSORT tool adapted for simulation studies [19] and the GREET Tool for educational studies [20].

The training took place during a mandatory one-day training course in emergency first aid organized for all 2nd-year medical students. One half-day was dedicated to placing someone in the recovery position and managing airway obstruction and first aid manoeuvrers in patients with ongoing limb or truncal haemorrhage. The other half-day was dedicated to BLS (i.e. CPR), during which the students practiced the procedure in dyads. This strategy is used in our institution [21] to increase the time of practice, practice relay between two rescuers and emphasize the need to communicate.

BLS was divided into five teaching steps which were taught step-by-step: (1) recognition of unconsciousness, (2) call for help, (3) recognition of the absence of breathing, (4) chest compressions and (5) use of a semi-automatic defibrillator. Two trained instructors (mostly anaesthesiologists) were involved each day and each of them supervised 10 students during practice periods. For each of the five steps, a first dyad practiced voluntarily in front of their observer counterparts and trainers. Time was then given to students to analyse performance, and to trainers to highlight correct practices, provide some pathophysiological explanations and disclose points for improvement. All dyads then consecutively practiced the resuscitation step(s) on the mannequin while the other students observed. At each time a dyad was formed, students had to practice with a student with whom they had not been in dyad before. Steps were incremented as they went along: (1) and (2), then (1), (2) and (3), then (1), (2), (3) and (4), and finally (1), (2), (3), (4) and (5). Each student was an active participant in the simulation once during each step, and observer several times per step.

A Resusci Anne Q-CPR mannequin Laerdal® was connected to an iPad® with the SkillReporter application from Laerdal®. Students were therefore provided real-time feedback information on the depth and rate of chest compressions, compression recoil, and the position of the student's hands on the mannequin's chest. An external cardiac massage effectiveness score was provided (percentage calculated using the proprietary Laerdal® algorithm). A semi-automatic training external defibrillator was also used.

**Fig. 1:**
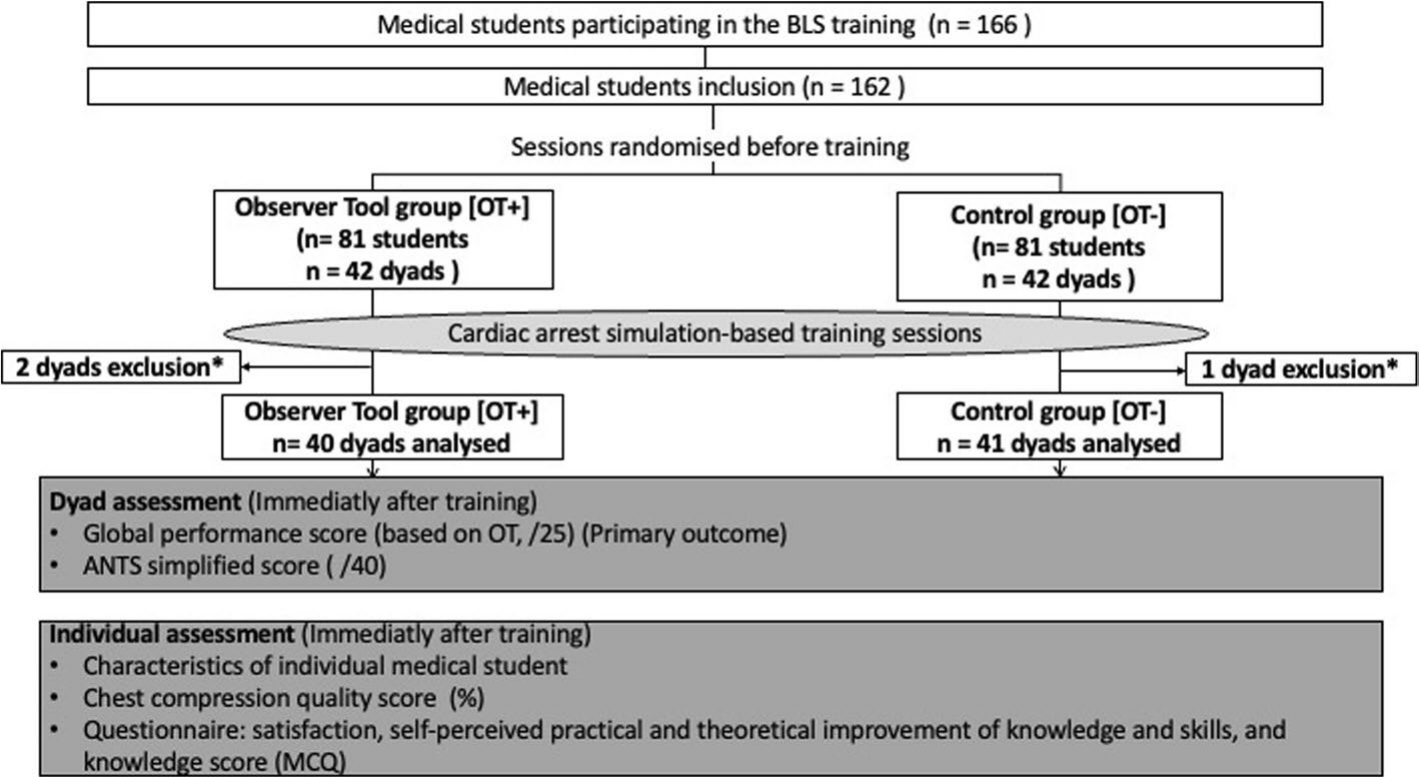
Flow chart. Criteria for non-inclusion in the study were inability to perform cardiopulmonary resuscitation on the mannequin (physical disability), unavailability for the end-of-day assessment or refusal to take part in the study. *Dyads were excluded from the analyses because the evaluation tool was incomplete

After written informed consent was obtained, medical students were included (Fig. 1). Randomisation was performed using the random function of Excel© software to obtain an equal number of sessions during which students used (OT +) or did not use (OT -) the observation tool.

The OT (Appendix 1) was designed by expert investigators in accordance with the 2021 recommendations of the European Resuscitation Council [22]. It was subsequently tested and validated by experts. In OT + groups, a form was given at the start of the BLS period of training, and students were instructed to fill out the form (observed/incompletely/not observed) for each of the 25 items and for each sequence in which they were observers. When only the first two steps were played, there were 7 items to check, and when further steps were played, there were 13, 20 and 25 items to check, respectively. The total number of items of the OT was therefore 25.

### Assessment method

Evaluation was performed at the end of the training day. For assessment, dyads were randomly composed of two students. The same mannequin as during the training was used, but no feedback was available. Students were told that results would be used for research purposes only. In the chosen scenario, they would manage together an out-of-hospital CPR, and they were asked to perform a 2-min scenario without interruption. The primary outcome was the global performance score of each dyad when performing the whole BLS process based on the OT checklist (each item being observed/not observed and noted 1/0 for a total score between 0 and 25) (level 2 of the Kirkpatrick model [23]).

The secondary outcomes included 1) a separate analysis of each item of the global performance score and 2) a simplified ANTS (Anaesthesia Non-Technical Skills) score ([24] evaluated by the evaluator and measuring the dyad’s performance using the four main categories (each scored out of 10): task management, teamwork, situational awareness and decision making and re-evaluation for a total score out of 40 (level 2 of the Kirkpatrick model). Although the ANTS score was used to assess jointly the two members of the dyad and elements of each category were not scored, its use was familiar to assessors who had used this scoring system in many previous sessions and studies. 3) Chest compression quality score (0–100%) provided by the SkillReporter® application (level 2 of the Kirkpatrick model). The SkillReporter® application from Laerdal® was validated by Davey et al. [25] and has been used in several previous studies [26, 27].

Students were invited to individually complete a questionnaire at the end of the training day that included several parts: 1) description of the student’s personal characteristics; 2) questions to assess their self-perceived practical and theoretical improvement of knowledge and skills using a Likert scale from 0 to 10 (level 2 of the Kirkpatrick model) and the change when compared with their perceived knowledge before the training day; and 3) their level of theoretical knowledge, based on a 10-question multiple-choice questionnaire (MCQ) (score out of 10) covering the major aspects of BLS (level 2 of the Kirkpatrick model).; 4) A satisfaction score about the training (Likert scale from 0 to 10) (level 1 of the Kirkpatrick model).

In addition, an OT compliance score for the intervention group was calculated by dividing the number of boxes filled in by each student by the theoretical total number of boxes.

### Statistical analysis

The global performance score (primary outcome) was compared immediately after training between the two groups (i.e., OT + versus OT-). Assuming an expected mean performance score of 18.5 out of 25 in the control group, using a standard deviation of 4 out of 25 points, and considering that an improvement of one standard deviation would be significant (difference accepted for studies in education [28]), a score of 22.5 out of 25 was expected in the intervention group. Using an alpha risk = 5% and a power of 90% with two-tailed tests, 27 dyads had to be included in each group to observe a significant difference https://biostatgv.sentiweb.fr/?module=etudes/sujets).

Secondary outcomes were also analysed by comparing the two groups immediately after training.

The results are presented as the median [25–75 interquartile] or percentage. Statistical analyses were carried out with the software GraphPad Prism^@^ (GraphPad Software, Inc., La Jolla, CA, USA). Statistical comparisons used a chi-square test for proportions and a Mann‒Whitney U test for nonparametric variables. A value of *p* < 0.05 was considered significant.

## Results

### Inclusion

In January 2022, 162 students were included after informed consent. They were randomised into two groups: 41 dyads in the intervention group (OT +) and 41 in the control group (OT-). After 3 dyad exclusions, 40 dyads were analysed in the intervention group (OT +) and 41 in the control group (OT-) (flow chart in Fig. 1). Participant characteristics were not different between the groups (Table 1).

**Table 1:** Characteristics of participants. Significant if *p*<0.05.Results presented as the median [25-75 interquartile] (Mann‒Whitney U test)

	OT+ (*n*=80)	OT- (*n*=76)	*p*
Age (median [25-75 interquartile]	19 [19-19]	19 [19-20]	0.87
Male (n (%))	19 (23)	19 (22)	0.84
Previous emergency care training (n (%)	9 (12)	8 (10)	0.71
Perception of theoretical knowledge *before training *(mean/10 ± SD)	7.5 [6-9]	8 [6-9]	0.40
Perception of practical knowledge *before training *(mean/10 ± SD)	7 [4-9]	7 [5-9]	0.66

Analysis of the observation forms completed by students in the OT + group showed that 59% of the OTs were fully completed.

### Global performance score for cardiopulmonary resuscitation (of the dyads)

**Fig. 2:**
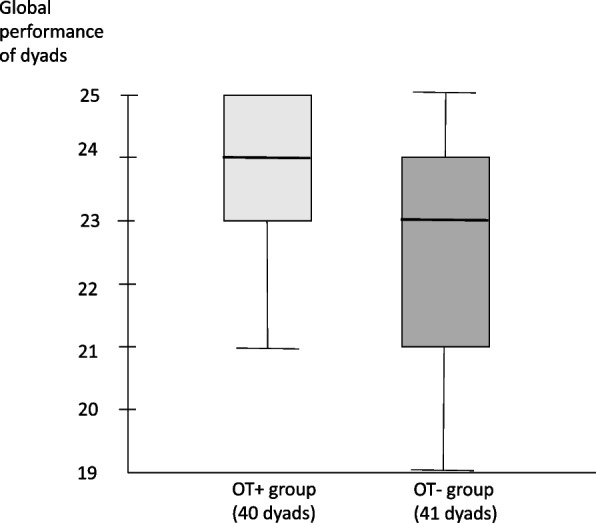
Comparison of global performance score items between the OT + and OT- groups (/25). Significant if *p* < 0.05. Results presented as the median [25^ème^-75.^ème^ interquartile] (Mann‒Whitney test). * *p*<0,05

Immediately after training, the global performance scores of the dyads were not significantly different between the OT + and OT- groups (Fig. 2). Secondary analysis showed a significant improvement in the scores for the "breathing" and "external cardiac massage" steps in the OT + group when compared with the OT- group (Table 2).

**Table 2:** Comparison of global performance score items between the OT+ and OT- groups. Significant if *p*<0.05.The results are presented as the median [25-75 interquartile] (Mann‒Whitney U test)

Score (per dyad)	OT+ (*n*=40)	OT- (*n*=41)	*p*
**Global performance (/25)**	24 [23 - 25]	23 [21 - 24]	0.052
**Recognition of unconsciousness (/3)**	3 [3 - 3]	3 [3 - 3]	0.109
**Call for help (/4)**	4 [4 - 4]	4 [4 - 4]	0,596
**Recognition of absence of breathing (/6)**	6 [5 - 6]	5 [5 - 6]	0,024*
**Chest compressions (/7)**	7 [5 - 7]	6 [5 - 7]	0.029*
**Use of a semi-automatic defibrillator (/5)**	5 [5 - 5]	5 [5 - 5]	1

* *p*<0.05

### Secondary outcomes

There was no significant difference between the two groups for the ANTS score or for the scores of perceived improvements of theoretical and practical skills (Table 3).

**Table 3:** Secondary outcomes. Significant if *p*<0.05.The results are presented as the median [25-75 interquartile] (Mann‒Whitney U test)

	OT+ (*n*=80)	OT- (*n*=76)	*p*
**ANTS simplified score (/40 points) (per dyad)**	35 (32-40)	33 (32-39)	0.60
**Chest compression quality score (%) (individual)**	76 (63-95)	89 (82-94)	0.37
**Knowledge score after the session (MCQ) (/10) (individual)**	8.0 (7.0-8,3)	6.7 (6.2-7.7)	0.048 *
**Difference in perceived theoretical knowledge (before-after) (individual)**	2 (1-4)	2 (1-3)	0.74
**Difference in perceived practical skills (before-after) (individual)**	2 (1-5)	3 (1-5)	0.97
**Satisfaction (/10) (individual)**	9 (8-10)	10 (9-10)	0.02 *

* *p*<0.05

The knowledge score (MCQ) was higher in the OT + group than in the OT- group (Table 3).

Students in the OT + group had significantly lower satisfaction scores than those in the OT- group (Table 3).

## Discussion

In this study, an OT based on BLS resuscitation steps was used with the aim of improving the training of medical students in CPR during a simulation session. To this end, we carried out a randomised controlled trial with one group using an OT (OT +) to be completed during training and a second control group without (OT-) during the same training. Use of this OT, however, was not associated with better global performance of medical students. However, secondary analysis showed that the use of OT improved two steps of CPR (i.e., assessment of breathing and performance of external cardiac massage). In addition, theoretical knowledge acquired immediately after training was significantly better in the OT + group, but satisfaction was higher in the group that did not use OT.

The number of learners in all healthcare professions has increased in our country in recent years and is planned to increase even more in the near future. Given the current capacities of simulation centers and the limited number of trained trainers, many sessions have a high number of learners, which means that not everyone can be an active participant. A recent meta-analysis showed that learning as an observer is probably less than that of an active participant in simulation sessions. [5]. Another potential usefulness of an OT is to use this tool also for students who can be active participants, with the aim of improving the learning outcomes above the level of the whole group above what is provided by the use of simulation itself. It is in this context that we tried to evaluate the use of an OT. In the present study indeed, all students were active participants several times during the session. The results were disappointing as they showed that using an OT does not increase the competency and knowledge level of our students above what was obtained in the group which did not use the OT. O’Regan et al. indeed proposed the use of OT [4], which is believed to allow more active learning by reinforcing attention during training and improving students’ performance [29]. The attention boost effect [30] is a theory that suggests that when two actions are performed simultaneously (in the present study, observing the other student and filling out the OT), attention is increased, and this would be even more true when the different elements to observe are frequent [31]. However, the available literature regarding the use of tools to increase observer learning is limited, heterogeneous and controversial, making interpretation still uncertain [8, 15–18]. Some studies have indeed shown a beneficial effect [15, 16]. One randomised study [16] howed an increased acquisition of medical knowledge and skills of anaesthesia residents when using an OT based on cognitive aids (i.e., emphasizing technical skills and medical knowledge). It is unclear as to why the OT produced a beneficial effect in this study and not in present one in which an OT was used to teach management of cardiac arrest. Several hypotheses can be put forward to explain the mixed results. In particular, it is possible that OTs may not have similar educational efficacy in various clinical circumstances. The most significant result was obtained in a study of technical skills (knowledge) in high-fidelity simulation [16]. In contrast, two randomised studies evaluating the effects of OT were unsuccessful at improving non-technical (in revision) or procedural skills (i.e. learning to insert a central line) (submitted). We expected to obtain good learning outcomes when teaching initial CPR, as it combines both technical skills (notably cardiac massage) and non-technical skills according to a highly standardized process, that is easy to describe in an OT. It is also of note that no guidelines on how to create and present an OT are available, and it remains possible that our OT was not easy to use.

It is also possible that our study was unable to demonstrate a beneficial effect, as the effect size in educational studies is often small. In all our studies, a reasonable power calculation was carried out beforehand, following the classic rule of using an expected effect size equal to the standard deviation of the overall effect [28]. However, estimating this standard deviation is tricky in the absence of prior data on which to rely. It is difficult to say whether the one-point difference (24 versus 23) in favour of OT use represents a real trend or the chance of measurement. It seems that only studies on a larger scale would enable us to clarify this point.

In this study, however, several secondary outcomes were associated with better performance when using OT. Some steps of CPR (assessment of respiratory status and cardiac massage) were better performed in the OT + group. These results suggest a potential benefit from the use of OT. Assessment of respiratory status involves at least two distinct, complementary and sequential steps: search for and removal of any upper airway obstruction followed by assessment of the respiratory rate. In our experience, the first part is often forgotten or incompletely carried out, as it seems to be less of a priority than calculating the frequency. Here, too, the iterative use during the session of the OT, which clearly mentions these two steps, could have improved acquisition and memorization so that their execution at the time of evaluation was more effective. Similarly, cardiac massage is a complex process to learn, as it needs to be performed in an extreme emergency situation, and its performance requires the conjunction of several simultaneous requirements (position of the rescuer, simultaneously providing adequate depth and frequency, etc.) which are difficult to achieve at the same time and with the same quality for each criterion. These requirements might overwhelm the cognitive capacities of the novice learner "who cannot think of everything at once". Using a cognitive aid in the form of an OT could have facilitated memorization and, secondarily, might have enabled the complete gesture to be performed more reflexively.

In this study, the OT + group performed significantly better on the knowledge score. Repeated reading of the items might have helped the students to clarify the concepts to be retained, explaining better results when the question was formally asked, although this was difficult to observe during the procedural evaluation. This result is in line with another study by our team [16], which also assessed both groups by MCQ at the end of training and showed better results in the group using an OT.

Satisfaction was very high in both groups, in line with the data of Seale et al. [18]. However, it was significantly lower in the OT + group. Limited compliance to filling in the tool (59%) is probably an indicator associated with lower satisfaction. Another possible reason is the cognitive pressure of having to fill in an OT.

The strengths of this study include the fact that our study was carried out randomly and prospectively. However, it also has several limitations. In all randomized studies described above, all participants had been active at least once on the simulator. Repetition and active participation might have improved learning and retention, explaining the very high scores achieved by students in the end-of-day assessment and therefore reducing the impact of OT. In addition, we assessed the students at the end of the day, a few hours after the initial training and not at a distance. In both groups, the performance scores were on average very high, close to the maximum score, whereas the expected average score of the control group had been estimated in the preliminary calculation at 18.5/25. These considerations may explain why no significant difference was found between the two groups. Ebbinghaus' forgetting curve [32] shows that to assess the effect of a teaching method on retention, it is necessary to measure students’ skills at a much greater distance. However, for logistical reasons, it was difficult to reconvene the students several months later.

Another limitation is that the ANTS score evaluates non-technical skills of a single actor [24] and other tools focusing on team functioning would probably have been more appropriate [33]. Finally, compliance with student completion of the OT was moderate (only 59%), although teachers insisted that the tool be completed by observers during each exercise. This incomplete use of OT may have diminished its impact on their learning. Several factors could have been involved: too long (with 25 items), too many repetitions were needed for students to maintain a continuous focus on the OT and the simulation they were watching, and it was probably difficult for students to watch, read and mark at the same time.

## Conclusions

The use of an OT did not show any pedagogical benefit on the global performance of CPR by second-year medical students immediately after simulation training. However, a potential benefit was found for several important secondary outcomes (learning to assess breathing, external cardiac massage performance and knowledge score). Further study is necessary to confirm these partially positive results.

### Supplementary Information

Supplementary Material 1.

## Data Availability

The datasets generated during and/or analysed during the current study are available in the figshare.com repository, 10.6084/m9.figshare.24720510.

## Abbreviations

ANTS: Anaesthesia non-technical skills
BLS: Basic life support
CPR: Cardiopulmonary resuscitation
OT: Observer tool
