# Supplementary material for: An observer tool to enhance learning of medical students during simulation training of cardiopulmonary resuscitation: a randomised controlled trial
Source: BMC Med Educ. 2024 Jul 3;24:719. doi: 10.1186/s12909-024-05658-x (PMC11223434; doi:10.1186/s12909-024-05658-x)
Supplement: Supplementary file 1 — Supplementary Material 1. [file 12909_2024_5658_MOESM1_ESM.docx]

**APPENDIX 1: Observer tool for CPR (medical student form**): four incremental sequences performed step-by-step

|  | 1 | 2 | 3 | 4 | 5 | 1 | 2 | 3 | 4 | 5 |
| --- | --- | --- | --- | --- | --- | --- | --- | --- | --- | --- |
| Items | Observed (O) / Incomplete (I) / Not observed (N) | | | | | | | | | |
| **Recognition of unconsciousness** |  | | | | | | | | | |
| He/she says: Mrs, Mr, open your eyes ! Shake my hands |  |  |  |  |  |  |  |  |  |  |
| He/she does nociceptive stimulation (nail pinch) |  |  |  |  |  |  |  |  |  |  |
| He/she announces loudly: "He/she is unconscious!” |  |  |  |  |  |  |  |  |  |  |
| **Call for help** |  | | | | | | | | | |
| He/she calls for help and contacts the emergency number by phone |  |  |  |  |  |  |  |  |  |  |
| He/she introduces himself and announces his/her phone number |  |  |  |  |  |  |  |  |  |  |
| He/she announces "The subject is unconscious” |  |  |  |  |  |  |  |  |  |  |
| He/she provides the exact location (street name and number, apartment number..) |  |  |  |  |  |  |  |  |  |  |
| **Recognition of the absence of breathing** |  | | | | | | | | | |
| He/she searchs for and remove any upper airway obstruction: foreign body, collar, tie, belt. |  |  |  |  |  |  |  |  |  |  |
| He/she places his/her cheek and counts (10 sec), aloud |  |  |  |  |  |  |  |  |  |  |
| He/she announces loudly "he's not breathing |  |  |  |  |  |  |  |  |  |  |
| He/she announces loudly   "He's in cardiac arrest” |  |  |  |  |  |  |  |  |  |  |
| He/she announces loudly the time |  |  |  |  |  |  |  |  |  |  |
| He/she requests "Please get a defibrillator". |  |  |  |  |  |  |  |  |  |  |
| **External cardiac massage** |  | | | | | | | | | |
| He/she starts massage (chest compression) within 2 minutes and continues until defibrillator is inserted |  |  |  |  |  |  |  |  |  |  |
| He/she positions himself/herself correctly: shoulders vertical over the chest, heel of the palm of both hands on the sternum at nipple level, arms straight and locked. |  |  |  |  |  |  |  |  |  |  |
| He/she counts out loudly, interspersing "And" between each number, and counts to 15 and repeats. |  |  |  |  |  |  |  |  |  |  |
| With a frequency between 100 and 120 |  |  |  |  |  |  |  |  |  |  |
| With a depth between 5 and 6 cm |  |  |  |  |  |  |  |  |  |  |
| He/she keeps the upper airway open |  |  |  |  |  |  |  |  |  |  |
| The relay between the 2 students is well executed |  |  |  |  |  |  |  |  |  |  |
| **Defibrillation** |  | | | | | | | | | |
| He/she positions patches correctly without interfering with massage |  |  |  |  |  |  |  |  |  |  |
| First-aiders massage until instructed to stop |  |  |  |  |  |  |  |  |  |  |
| He/she delivers a shock if advised by the defibrillator |  |  |  |  |  |  |  |  |  |  |
| After shock, he/she resumes massage for 2 min, and follows instruction) (relay between rescuers) |  |  |  |  |  |  |  |  |  |  |
| After the 2nd defibrillator evaluation, he/she takes the pulse if no shock is mandated. |  |  |  |  |  |  |  |  |  |  |


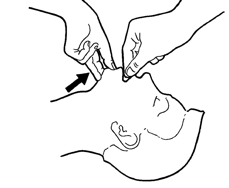

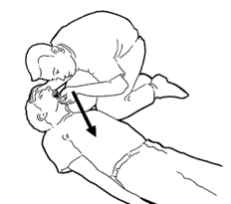

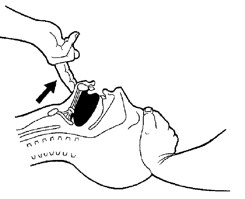

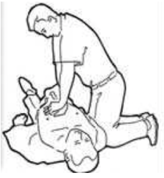

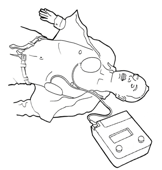


|  | 1 | 2 | 3 | 4 | 5 | 1 | 2 | 3 | 4 | 5 |
| --- | --- | --- | --- | --- | --- | --- | --- | --- | --- | --- |
| Items | Observed (O) / Incomplete (I) / Not observed (N) | | | | | | | | | |
| **Recognition of unconsciousness** |  |  |  |  |  |  |  |  |  |  |
| He/she says: Mrs, Mr, open your eyes ! Shake my hands |  |  |  |  |  |  |  |  |  |  |
| He/she does nociceptive stimulation (nail pinch) |  |  |  |  |  |  |  |  |  |  |
| He/she announces loudly: "He/she is unconscious! |  |  |  |  |  |  |  |  |  |  |
| **Call for help** |  |  |  |  |  |  |  |  |  |  |
| He/she calls for help and contacts the emergency number by phone |  |  |  |  |  |  |  |  |  |  |
| He/she introduces himself and announces his phone number |  |  |  |  |  |  |  |  |  |  |
| He/she announces "The subject is unconscious” |  |  |  |  |  |  |  |  |  |  |
| He/she provides the exact location (street name and number, apartment number..) |  |  |  |  |  |  |  |  |  |  |
| **Recognition of the absence of breathing** |  |  |  |  |  |  |  |  |  |  |
| He/she searchs for and remove any upper airway obstruction: foreign body, collar, tie, belt. |  |  |  |  |  |  |  |  |  |  |
| He/she places his/her cheek and counts (10 sec), aloud |  |  |  |  |  |  |  |  |  |  |
| He/she announces loudly "he's not breathing |  |  |  |  |  |  |  |  |  |  |
| He/she announces loudly   "He's in cardiac arrest” |  |  |  |  |  |  |  |  |  |  |
| He/she announces loudly  the time |  |  |  |  |  |  |  |  |  |  |
| He/she requests "Please get a defibrillator" |  |  |  |  |  |  |  |  |  |  |
| **External cardiac massage** |  |  |  |  |  |  |  |  |  |  |
| He/she starts massage (chest compression) within 2 minutes and continues until defibrillator is inserted |  |  |  |  |  |  |  |  |  |  |
| He/she positions himself/herself correctly: shoulders vertical over the chest, heel of the palm of both hands on the sternum at nipple level, arms straight and locked. |  |  |  |  |  |  |  |  |  |  |
| He/she counts out loud, interspersing "And" between each number, and counts to 15 and repeats. |  |  |  |  |  |  |  |  |  |  |
| With a frequency between 100 and 120 |  |  |  |  |  |  |  |  |  |  |
| With a depth between 5 and 6 cm |  |  |  |  |  |  |  |  |  |  |
| He/she keeps the upper airway open |  |  |  |  |  |  |  |  |  |  |
| The relay between the 2 students is well executed |  |  |  |  |  |  |  |  |  |  |
| **Defibrillation** |  |  |  |  |  |  |  |  |  |  |
| He/she positions patches correctly without interfering with massage |  |  |  |  |  |  |  |  |  |  |
| First-aiders massage until instructed to stop |  |  |  |  |  |  |  |  |  |  |
| He/she delivers a shock if advised by the defibrillator |  |  |  |  |  |  |  |  |  |  |
| After shock, , he/she massages for 2 min and follows instructions (resumes massage) (relay between rescuers) |  |  |  |  |  |  |  |  |  |  |
| After the 2nd defibrillator evaluation, he/she takes the pulse if no shock is mandated. |  |  |  |  |  |  |  |  |  |  |


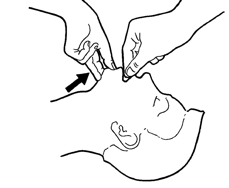

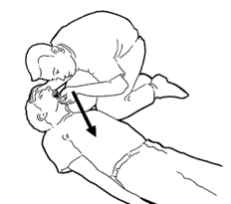

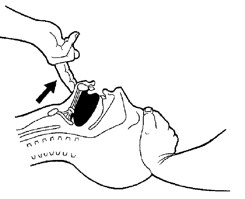

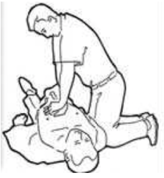

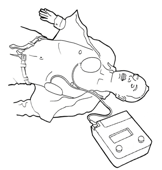


**APPENDIX 2** : simplified ANTS score of the dyad. Evaluation at the end of the session.

Each behaviour scored by the evaluator between 0 and 10, then added to form a global ANTS score with a maximum value of 40.

| **Behaviour assessed** | **Note between 0-10** |
| --- | --- |
| Task Management |  |
| Team Working |  |
| Situation Awareness |  |
| Decision Making |  |
